# Engineering psychiatric education 2.0 in post-pandemic europe

**DOI:** 10.1192/j.eurpsy.2021.192

**Published:** 2021-08-13

**Authors:** L. De Picker

**Affiliations:** Sinaps, University Psychiatric Hospital Campus Duffel, Duffel, Belgium

**Keywords:** trainees, Medical Education, innovation, postgraduate psychiatric training

## Abstract

In the world of medical education, there is generally a lot of emphasis on following procedure and tradition, and a reluctance to challenge the norm. It takes insight and courage to question traditional approaches and paradigms: why are psychiatric training programs done the way that they are? Reverse engineering involves taking something apart and analysing its workings to figure out how it does what it does and how it can be improved. In education, reverse engineering implies one determines learning outcomes upfront and then works back from them. Applied to postgraduate psychiatric training, it requires us to determine the basic principles or core concepts resulting in the successful formation of a well-rounded psychiatrist. In times of crisis there usually is more leeway to challenge the status-quo – hence the saying “never waste a good crisis”. Indeed, if the COVID-19 crisis has taught us anything, it is that education should be meeting learners where their attention is at, and that any healthcare organisation can be transformed within weeks when given the right incentives. In this workshop, Dr. De Picker will reflect on how post-COVID European psychiatric training can reinvent itself to address long-standing concerns and unmet needs. Innovative approaches will be needed to start shaping the psychiatrists of the future.

**Disclosure:**

No significant relationships.

